# COVID-19 Reflections on Restorative Treatments of Permanent First Molars

**DOI:** 10.7759/cureus.36394

**Published:** 2023-03-20

**Authors:** Zeynep Ceren Celik, Cigdem Elbek Cubukcu

**Affiliations:** 1 Restorative Dentistry, Bursa Uludağ University, Bursa, TUR; 2 Pedodontics, Bursa Uludağ University, Bursa, TUR

**Keywords:** restorative dentistry, dental procedures, composite resin, dental amalgam, first permanent molar, covid-19

## Abstract

Background and Aim: It is important to provide appropriate dental care for newly erupted permanent first molars (PFMs) since they are susceptible to caries. As the coronavirus disease 2019 (COVID-19) pandemic has led to significant changes in the way dental services are provided to patients, the purpose of this study is to examine the procedure records assigned to PFMs of 6-15 year-olds during the pandemic and analyze the restorative material preferences of the residents of public dental hospitals.

Materials and Methods: Procedure records of patients aged between 6-15 years were extracted from the Public Oral and Dental Health Center, Bursa, Türkiye. All teeth groups except PFMs were excluded, while extracted, survived (restorative/endodontic/prosthetic procedures), and prevented (fissure sealant application) PFMs were analyzed retrospectively. Furthermore, restorative material preferences were analyzed by arch location, cavity surfaces, and dentition types.

Results: Strong positive correlation was seen between age and PFM extraction (r=0.973; p<0.001) and age and PFM restorative treatments (r= 0.966; p<0.001); a negative correlation was detected between age and fissure sealants (r= -0.984; p<0,001) performed on PFMs of 8-15-year-olds. Amalgam was most often preferred as the restorative material (p<0.05).

Conclusion: The distribution of treatments and dental restorations can vary based on many factors, and the pandemic conditions may have changed treatment preferences to favor preventive dentistry. The excess of multi-surface restorations may be related to the delay of treatment applications during COVID-19.

## Introduction

Permanent first molars (PFMs) have been identified as the most caries-prone teeth in permanent dentition. This is likely due to their location at the back of the dental arch, making them difficult to reach with a toothbrush, as well as their earlier emergence in the oral cavity compared to the other permanent teeth [1].

According to the literature, 15.9-49% of children between 6-17 years of age have experienced dental caries (tooth decay) in their PFMs [1-4]. PFMs are very important because of their significant role in maintaining normal masticatory function and dentofacial harmony [5]; therefore, their effect on the overall development of children cannot be overemphasized. However, it is the very lack of visibility for molars compared to the anterior teeth, combined with the increased difficulty of cleaning the posterior interdental spaces, which increases the caries risk of PFMs. Therefore, during childhood [6], the prevention of FPM caries plays a critical role in maintaining good oral hygiene habits, such as regular brushing and flossing as well as visiting a dentist regularly for checkups and cleanings. Additionally, dental sealants are a preventive measure that can be applied to the chewing surfaces of PFMs to protect them from decay [2].

Since March 11, 2020, dental offices have implemented various infection control protocols to minimize the risk of coronavirus disease 2019 (COVID-19) transmission, including the use of personal protective equipment (PPE), screening patients for symptoms, and implementing social distancing measures. Therefore, patients should feel confident in seeking dental treatment if they experience dental caries or other oral health issues. According to the circular published by the Republic of Türkiye Ministry of Health, the postponement of aerosol-generating non-emergency treatments in all health institutions, including dental practices, clinics, and centers was highly recommended [7].

The delivery of dental services during the COVID-19 pandemic may have been influenced by various additional factors other than the availability of dental services, such as patient preferences, the severity of dental caries, the presence of dental pain, etc. Some patients may prefer non-invasive treatments, such as fluoride treatment or dental sealants over more invasive procedures such as fillings or extractions, to minimize the risk of exposure to COVID-19. However, the choice of treatment will depend on the severity of the caries and the dentist's professional judgment [8].

Therefore, the aims of this study are to examine dental treatments performed on PFMs during the COVID-19 pandemic and analyze the restorative material preferences of the residents of the Public Dental Hospital in Bursa, Türkiye.

## Materials and methods

Ethics

This study was approved by the Faculty of Medicine Clinical Research Ethics Committee of Bursa Uludag University (approval number: 2021-12/25 dated September 8, 2021). Legal permission was obtained from the Ministry of Health COVID-19 Scientific Research Platform (No: 2021-08-26T13_55_16).

Design

**Figure 1:**
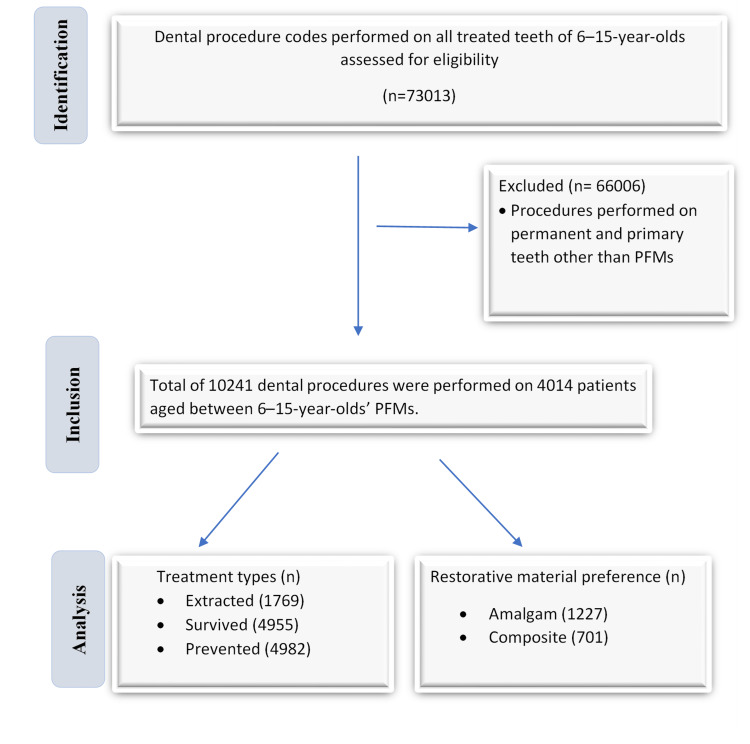
Flowchart of the methodology of the present study. PFM: permanent first molar

Data of the 6-15-year-old patients from January 1, 2020, to December 31, 2020, were derived from the Bursa Oral and Dental Health Training and Research Hospital patient registration software (Trtek Web Patient Information Management System ver. 10.0.795, TRtek Yazılım Ltd., Gölcük, Türkiye) and reviewed retrospectively. The focus was PFMs and other groups of teeth were excluded. PFM extractions, dental treatments (restorative, endodontic, and prosthodontic), and fissure sealants were coded as extracted, survived, and prevented, respectively (Figure 1). Restorative material preferences were analyzed by cavity surfaces (single/multi) and localization (maxilla/mandibula). The flowchart of the present study is given in Figure 1.

Statistical analysis

The results are presented as frequencies and percentages. Categorical variables were compared between the groups using Pearson’s chi-square test and Fisher’s exact test, while the Bonferroni test was used for multiple comparisons. Pearson's correlation was applied in measurements of the statistical relationship between age groups and procedures (n) variables. Statistical significance was set at p < 0.05, and statistical analyses were performed using IBM SPSS Statistics for Windows, Version 23.0 (Released 2015; IBM Corp., Armonk, New York, United States).

## Results

The results indicated that sex distribution (female/male) was homogeneous with a ratio of 2135/1879. The number of patients of all ages and the number of treatment procedures were comparable (Table 1). As indicated in Table 1, the number of treatments increased while preventive measures (fissure sealants) decreased with age. Fissure sealants were applied during the COVID-19 pandemic.

**Table 1:** Extractions, dental treatments, and fissure sealent applications performed on PFMs of 6-15-year-olds PFM: permanant first molar

Age	Patients (N)	Procedures (N)	Extracted PFMs n(%)	Survived PFMs (Dental treatments) n(%)	Prevented PFMs (Fissure sealent) n(%)
6	124	378	0 (0)	26 (6.9)	351 (93.1)
7	234	768	12 (1.5)	77 (10.0)	619 (80.6)
8	333	1006	17 (1.7)	155 (15.4)	825 (82.0)
9	351	998	40 (4.0)	249 (24.9)	709 (71.0)
10	400	1124	65 (5.8)	430 (38.3)	629 (55.9)
11	504	1431	91 (6.3)	740 (51.7)	601 (41.9)
12	479	1274	115 (9.0)	673 (52.8)	487 (38.2)
13	515	1267	152 (11.9)	833 (65.7)	284 (22.4)
14	537	1331	138 (10.4)	918 (68.8)	277 (20.8)
15	537	1192	139 (11.6)	854 (71.6)	200 (16.8)

In all age groups in the study, amalgam restorations were preferred on mandibular PFMs regardless of single- or multi-surface cavities. Table 2 shows that multi-surface restorations were performed significantly more in both children with mixed dentition (6-11 years) and permanent dentition (12-15 years) (p<0.05). In addition, it has been observed that amalgam restorations (n=579) were more numerical with no significance comparing composites (n=300) in 6-11-year-olds (p=0.107). Whereas, a significance was observed in favor of amalgam multisurface restorations performed on 12-15-year-olds' PFMs (p=0003). Except for single surface restorations on 12-15-year-olds’ PFMs, all restorative treatments were significantly higher in mandibular arches (Table 2).

**Table 2:** Restorative treatment details of 6-11-year-olds (mixed dentition) and 12-15-year-olds (permanent dentition)

Cavity Surfaces	Single-surface				Multi-surface			
Restorative Material	Amalgam		Composite		Amalgam		Composite	
Localization of the restorations	Maxilla	Mandibula	Maxilla	Mandibula	Maxilla	Mandibula	Maxilla	Mandibula
Age groups								
6-11 (n=879)	72 (29.4)	173 (70.6)	63 (43.8)	81 (56.3)	110 (32.9)	224 (67.1)	53 (34.0)	103 (66.0)
p	<0.001		0.134		<0.001		<0.001	
12-15 (n=1029)	58 (43.0)	77 (57.0)	58 (43.9)	74 (56.1)	184 (35.9)	329 (64.1)	108 (40.1)	161 (59.9)
p	0.102		0.164		<0.001		0.001	

**Figure 2:**
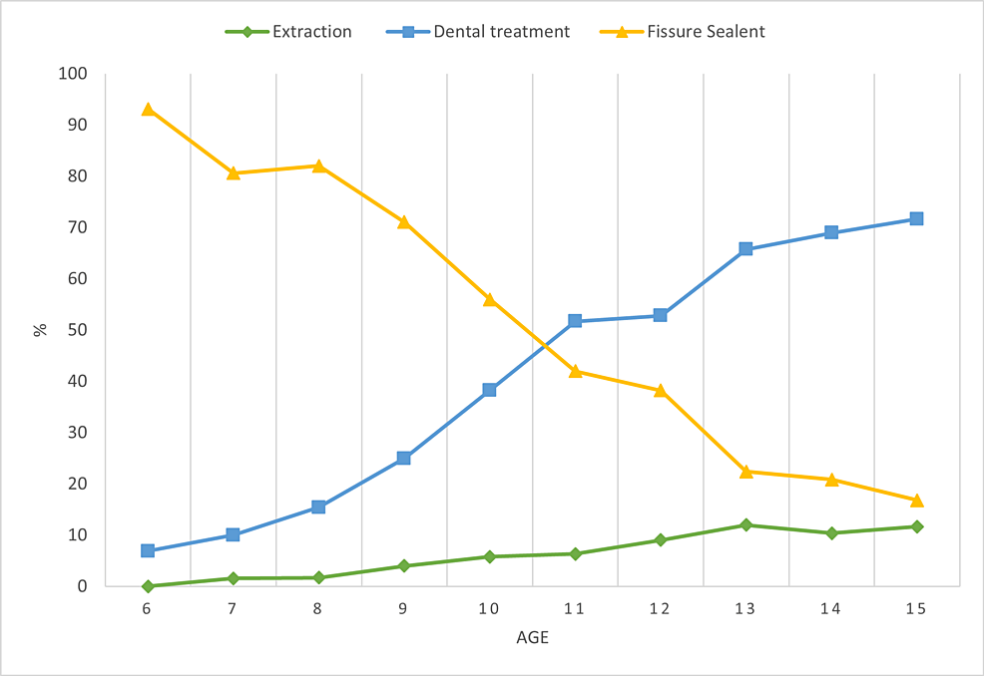
Percentage distribution of the procedures (extractions, dental treatments, fissure sealants) by age

Very strong positive correlations between age and PFM extraction (r=0.973; p<0.001) and age and. PFM restorative treatments (r= 0.966; p<0.001) were detected. A moderate negative correlation was seen between age and fissure sealant with no significance (r= -0.623; p= 0.054). Excluding the data of the ages of 6 and 7 years for fissure sealants, a strong negative correlation was detected between age and fissure sealants (r= -0.984; p<0.001) (Figure 2).

## Discussion

The permanent first molars, also known as the six-year molars, are the teeth that are most commonly affected by dental caries [2-4,9]. Consistent with the results of a recent study investigating a similar age group [10] as this research project, 14% of all dental procedures were performed on PFMs and showed no sex difference. 

Despite the COVID-19 pandemic conditions, it was noticeable that dentists performed higher rates of fissure sealants as a non-aerosol-generating preventive measure to sound PFMs [11].

In accordance with the literature [12,13], a decrease in the number of fissure sealants and an increase in the number of restorations are observed with increasing age. This situation can be associated with the eruption of the second molar in the mouth over time and poorer oral hygiene practices.

It is possible that postponing non-emergency dental treatments during the COVID-19 pandemic may have led to an increase in the severity of dental problems when patients were finally able to seek treatment [14,15]. The lockdown processes and measures to prevent horizontal transmission of the disease resulted in many dental offices closing or limiting their services to emergency care only; this meant that patients with non-emergency dental issues, such as initial caries lesions, may have had to delay their treatment for several months. In this study, the number of multi-surface dental restorations in both age groups (6-11 years and 12-15 years) was found to be statistically higher than in single-surface restorations. 

Multi-surface restorations, regardless of restorative material, were more commonly applied to the mandibular PFMs in both the 6-11 years and 12-15 years age groups, which is consistent with a recent clinical study investigating the caries prevalence and associated risk factors in the PFMs of 11-14-year-olds [9]. However, the results of a clinical study conducted in the Turkish population for older ages found the distribution was higher in the maxilla (62.4%) than in the mandibula (37.6%) [16]. Nevertheless, it is important to note that the distribution of dental restorations can vary based on many factors, including the individual patient's oral health, diet, and dental hygiene practices, as well as the preferences and clinical judgment of the treating dentist.

When the choice of restorative material was evaluated, amalgam was preferred in all groups except single-surface restorations in the 12-15 years age group. This result may be related to patient cooperation, which is a crucial parameter regarding the application steps of dental composite restorations [17]. This result also might be associated with the survival rate of these dental materials, thus a recent study suggests higher survival rates of amalgam restorations (94.4%) the composite restorations (85.5%) performed on 8-12-year-olds' posterior teeth [18]. Additionally, for amalgam restorations, the annual failure rates ranged from 0.16% to 2.83%, while for composite restorations, they ranged from 0.94% to 9.43% [18]. This outcome, suggests that composite restorations are more likely to fail within a shorter period than amalgam restorations which may be disadvantageous in a period of pandemic. Similarly according to Mjör [19]. who investigated failure rates of composite and amalgam restorations, the median survival age for composite was six years, whereas it was nine years for amalgam.

According to the literature, the mandible typically bears more occlusal forces than the maxilla as the occlusal pressure was measured as 68·3 ± 22·9 MPa at maxillary molars; and 69·2 ± 27·6 MPa in mandibular molars [20]; this results in an increased force on the lower teeth which may make them more prone to dental decay or other types of damage that require multi-surface restorations, such as large cavities or fractures. Additionally, the lower molars often have a more complex anatomy than the upper ones, with more grooves and fissures which might be retentive for food particles and bacterial products, rendering them more susceptible to caries formation and making it more difficult to protect them with routine oral hygiene measures [21].

Amalgam and composite resin are commonly used restorative materials for PFMs [22]. While amalgam is a durable material that has been used for decades, it is not esthetically pleasing; in contrast, composite resin is more esthetically pleasing but may not be as durable as amalgam [22].

Limitations

The main limitation of the study is the limited information about patients' present caries experiences in PFMs, their intraoral conditions, and the condition of the teeth that were not included in the extracted, survived, or prevented categories. Thus, the present study is a cross-sectional analysis based on a retrospective examination of dental records; diagnostic records would provide an opportunity for a broader analysis.

## Conclusions

The choice of restorative material for PFMs should be made on a case-by-case basis, taking into account the extent of the decay, the patient's preference, and the dentist's experience and expertise with the different materials. In addition to choosing the appropriate restorative material, it is important to maintain good oral hygiene practices and attend regular dental check-ups to prevent further decay and preserve the longevity of the restoration. During COVID-19, amalgams may have been preferred due to their advantages, such as shorter clinical application times, higher survival rates, and low repetitions due to adhesive failures.
